# Vaginal Administration of Progesterone in Twin Gestation: Influence on Bone Turnover and Oxidative Stress

**DOI:** 10.3390/antiox14030324

**Published:** 2025-03-08

**Authors:** María Puche-Juarez, Juan M. Toledano, Jorge Moreno-Fernandez, Javier Diaz-Castro, Javier Sánchez-Romero, María Mar Gil, Valeria Rolle, Aníbal Nieto-Díaz, Julio J. Ochoa, Catalina De Paco Matallana

**Affiliations:** 1 Department of Physiology, Faculty of Pharmacy, Campus Universitario de Cartuja, University of Granada, 18071 Granada, Spain; mpuchej@ugr.es (M.P.-J.); javierdc@ugr.es (J.D.-C.); 2 Institute of Nutrition and Food Technology “José Mataix Verdú”, Biomedical Research Centre, University of Granada, 18016 Armilla, Spain; 3 Nutrition and Food Sciences Ph.D. Program, University of Granada, 18071 Granada, Spain; 4 Instituto de Investigación Biosanitaria (IBS) (E15-EXPODIET; MP19), 18016 Granada, Spain; 5 Department of Obstetrics and Gynecology, ‘Virgen de la Arrixaca’ University Hospital, 30120 Murcia, Spain; javier.sanchez14@um.es (J.S.-R.); anibal.nieto@um.es (A.N.-D.); katy.depaco@gmail.com (C.D.P.M.); 6 Faculty of Medicine, Universidad de Murcia, 30120 Murcia, Spain; 7 Institute for Biomedical Research of Murcia, IMIB-Arrixaca, El Palmar, 30120 Murcia, Spain; 8 Department of Obstetrics and Gynecology, Hospital Universitario de Torrejón, 28850 Torrejón de Ardoz, Spain; mmar1984@gmail.com (M.M.G.);; 9 Faculty of Medicine, Universidad Francisco de Vitoria, 28223 Pozuelo de Alarcón, Spain

**Keywords:** twin pregnancy, progesterone, bone turnover, oxidative stress

## Abstract

Twin pregnancies, with higher incidences of preterm birth, are becoming more prevalent. Progesterone has shown effectiveness in the prevention of preterm labour, though other factors related to pregnancy and neonatal health may be affected by this hormone and have not been previously addressed. This study aims to evaluate the impact of progesterone administration on oxidative stress and bone turnover during twin gestation and investigate associations with some maternal/neonatal variables of interest. Women pregnant with twins were recruited in the “Virgen de la Arrixaca” University Hospital and randomly assigned to two groups: control (n = 49) and progesterone (n = 50). A total of 600 mg/day of progesterone was vaginally administered from 11 to 14 to 34 weeks of gestation. Blood samples were taken in the first (T1) and third trimester (T3), analyzing biomarkers related to oxidative stress and bone turnover. Most bone turnover and oxidative markers experiment with significant changes during gestation. Progesterone administration significantly increased (*p* < 0.05) the levels of osteocalcin in T3 and decreased (*p* < 0.05) the levels of sclerostin. Regarding oxidative stress, the progesterone group, unlike the control group, showed no significant increase in oxidative stress between T1 and T3. In conclusion, results show that progesterone administration could increase maternal bone formation and modulate oxidative stress.

## 1. Introduction

Over the past few decades, the prevalence of multiple pregnancies, particularly twin pregnancies, has significantly increased, now accounting for 3–4% of all pregnancies in developed countries, largely due to the widespread use of assisted reproductive technologies [1,2]. These kinds of pregnancies are associated with increased risk of neonatal morbidity and mortality [3], long-term neurodevelopmental disability [4] and increased maternal complications such as hypertensive disorders [5]. In addition, the risk of preterm birth in twin pregnancies is approximately ninefold higher compared to singleton pregnancies, making preterm delivery a major contributor to the increased morbidity and mortality observed in twins [6,7]. In order to reduce this high incidence of preterm birth, various interventions have been implemented, although their outcomes have often fallen short of expectations [8]. Among these interventions, the administration of progesterone has emerged as a potential strategy to mitigate preterm birth risks.

Progesterone is a steroid hormone capable of regulating a wide variety of physiological processes in multiple tissues, although it has also been implicated in the development of conditions such as breast cancer [9]. In pregnancy, progesterone plays a crucial role in maintaining uterine quiescence, a key factor in sustaining gestation [10]. In this sense, a recognized hypothesis is the “Progesterone block hypothesis” which indicates that this steroid hormone maintains pregnancy and prevents preterm birth [9]. Different studies have shown the efficacy of the administration of progesterone in preterm birth [11]. Its supplementation in the second and third trimester of pregnancy essentially decreases the risk of preterm birth in high-risk singleton pregnancies, including cases with a short cervix, at 23 weeks of gestation [9,12,13,14]. Although the precise mechanisms by which progesterone prevents preterm birth are not fully understood, it is believed to reduce maternal inflammation in the decidua and cervix [10,15]. For these reasons, vaginal progesterone administration is widely recommended for pregnancies at high risk of preterm delivery. However, we cannot forget that this is a hormone with important physiological effects that could influence pregnancy and maternal–fetal health, such as, for example, effects related to bone turnover or oxidative stress, which warrant further investigation [16,17].

Bone turnover is significantly elevated during pregnancy to meet the demands of constructing a new skeleton, which necessitates substantial calcium transfer to the developing fetus. This would lead to maternal skeleton deterioration unless powerful compensatory mechanisms come into play to achieve an equilibrium between absorption and resorption processes [18]. Pregnancy-related changes may cause a deterioration in mothers’ bone mass density, with an increased risk of fracture and osteoporosis, even if they are transitory changes [19]. In twin pregnancies, these effects are often more pronounced due to the greater calcium demand from the fetuses [18]. Given the potential implications for maternal health, fetal development, and neonatal outcomes, studying potential factors that may affect bone turnover during pregnancy is critically important. In this sense, the study of bone turnover during pregnancy requires consideration of various factors that can influence this process, such as the physiological link between bone and energy metabolism, in which osteocalcin, leptin, and insulin are involved [20,21]. Additionally, oxidative stress, which is heightened during pregnancy, plays a significant role in bone turnover [22]. In addition, this factor has been shown to be crucial in the pathophysiology of several pregnancy complications, including miscarriage, pre-eclampsia, intrauterine growth restriction (IUGR), and preterm birth [23].

As previously noted, another crucial factor to consider is the administration of progesterone, particularly in pregnancies at high risk for preterm delivery, where its use is frequently recommended. Progesterone has a key role in mineral and skeletal metabolism. This steroid hormone is important in activating skeletal remodelling and coupling bone resorption with bone formation [16]. Progesterone serves as an intriguing link between the ovaries and bone health in women. Several studies have demonstrated its role in preventing and treating osteoporosis by promoting bone formation through direct stimulation of osteoblast activity [24,25]. However, most research has focused on progesterone supplementation during menopause, with limited studies on its effects in pregnant women or newborns, and an absence of research specifically addressing twin pregnancies [24,25,26]. This group is of special interest due to its higher bone turnover [18], combined with the increasing focus on using progesterone to reduce preterm birth in this population [14]. In addition, progesterone has shown a clear effect on oxidative stress, a factor of great importance in the normal development of pregnancy and related to bone turnover, as mentioned above. Several studies show a reduction in lipoperoxidation and oxidative stress by this hormone, either through a reduction in the production of free radicals, for example, improving mitochondrial functionality, or through an increase in the activity of the antioxidant defence system, with most studies also showing a dose-dependent antioxidant effect [9,17,27]. Even though a prooxidant effect has also been observed in some studies [28].

As indicated above, the implications of progesterone administration in twin pregnancies on bone turnover and oxidative status have never been investigated before and, given the importance of these two factors in maternal, fetal, and neonatal health, it is necessary to further investigate these effects. Consequently, the objective of this study is to assess the effect of the vaginal administration of 600 mg of progesterone during twin gestation on bone turnover and oxidative stress during pregnancy, and to explore the °1existence of relationships between the main maternal and neonatal clinical outcomes with the biomarkers studied.

## 2. Materials and Methods

### 2.1. Subjects

**Figure 1:**
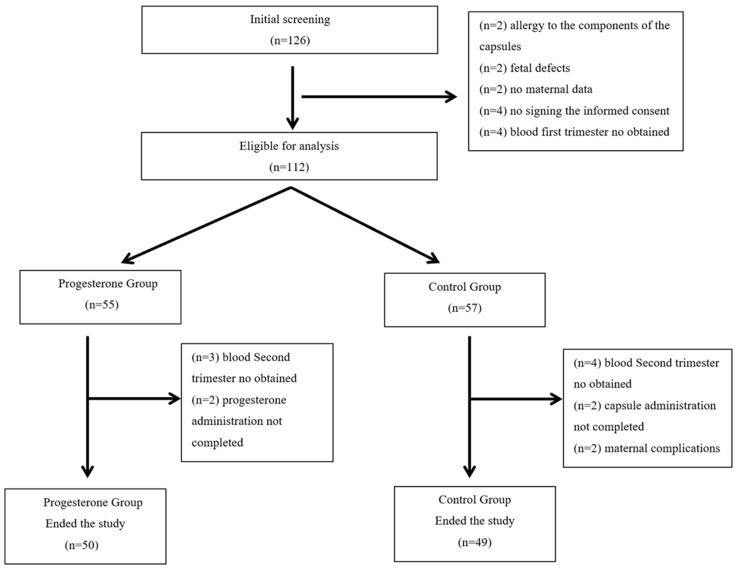
Flowchart showing participant enrollments and dropouts.

This is a secondary, post hoc, study carried out within the EVENTS study (early vaginal progesterone for the prevention of spontaneous preterm birth in twins: A randomized, placebo-controlled, double-blind trial). The trial is registered in the European Union Drug Regulating Authorities Clinical Trials database (EudraCT number 2015-005180-16) and with ISRCTN (ISRCTN66445401). The primary study was a randomized, placebo-controlled, double-blind trial carried out in 22 hospitals and whose main outcome was to verify if, among twin pregnancies, vaginal administration of progesterone at a dose of 300 mg twice per day from 11 to 14 weeks to 34 weeks of gestation may decrease the incidence of preterm delivery before 34 weeks of gestation. In the present study, only samples recruited at the “Virgen de la Arrixaca” University Clinical Hospital in Murcia (Spain) were used. All hospitals involved in the primary study, including this hospital, followed the same experimental design, recruitment process, randomization and masking, and procedure, which have been previously published [15]. Briefly, the mothers, with twin pregnancies, were recruited during the 11^+0^ to 13^+6^ weeks in the hospital; the conditions of the study were informed and, after the informed consent was accepted and signed, they began the randomized assigned process, in a 1:1 ratio, to one or the other of the study groups. The flowchart for participant enrolment and drop-outs is shown in Figure 1.

The progesterone group (n = 50) received information on how to self-administer a vaginal capsule twice a day (600 mg total) throughout the study, ending this vaginal insertion at 34 weeks of gestation or earlier in case of preterm birth. The control group (n = 49) performed the same process but with capsules containing a placebo. The placebo and progesterone capsules were identical except for the presence or absence of progesterone. Both capsules were supplied by Besins Healthcare, Brussels, Belgium.

The following inclusion criteria were used: older than 18 years, twin pregnancy, presence of 2 fetuses in the ultrasound from week 11 to 13, and informed and written consent. The exclusion criteria used were: monoamniotic pregnancies, early signs of Twin-Twin Transfusion Syndrome, presence of significant fetal anomalies, nuchal translucency ≥3.5 mm, women with physical (hepatic dysfunction, thromboembolic disorder, carcinoma, etc.) or severe mental illness, hypersensitivity to progesterone or regular treatment with progesterone in the days prior to the start of the study, and allergy to the components of the capsules (sunflower oil, soy, lecithin, etc.). Several visits and interviews were carried out during pregnancy and after delivery, obtaining maternal, fetal and neonatal data, such as maternal age, weight and height, type of conception, parity, chorionicity, cervical length, preeclampsia, length of the larger fetus, prematurity (before 37 weeks of gestation), gestational age at delivery, birth weight, neonatal therapy (admission to NICU, need for ventilation), or neonatal morbidity (Respiratory distress syndrome; Intraventricular hemorrhage; Anemia; Necrotizing enterocolitis; Retinopathy; Sepsis). The study was conducted in accordance with the Declaration of Helsinki, and the protocol was approved by the Ethics Committee of the “Virgen de la Arrixaca” University Clinical Hospital (Murcia, Spain) (2021-4-5-HCUVA).

### 2.2. Blood Sampling

Venous blood (5.0 mL) was collected in the first trimester (T1) between 11 and 14 weeks, before starting progesterone treatment and in the third trimester (T3) at week 32, after finishing progesterone treatment in a serum vacutainer tube with separating gel. After sitting for 30 min at room temperature, the blood was centrifuged (3000 rpm during 10 min) and the serum obtained was frozen (−80 °C) for further analysis.

### 2.3. Bone Turnover

Parathyroid hormone (PTH), osteocalcin (OC), osteopontin (OPN), osteoprotegerin (OPG), sclerostin (SOST), DKK1, IL-6, TNF-α, insulin, and leptin were determined using the HBNMAG-51K MILLIPLEX MAP Human Bone Magnetic Bead Panel, based on immunoassays on the surface of fluorescent-coded beads (microspheres), following the specifications of the manufacturer (50 events per bead, 50 µL sample, gate settings: 8000–15,000, time out 60 s). The Protein Receptor Activator for Nuclear Factor B Ligand (RANKL) was determined using the MILLIPLEX Human RANKL Single Plex Kit, following the specifications of the manufacturer. The plates were read on a LABScan 100 analyzer (Luminex Corporation, Austin, TX, USA) with xPONENT 4.3 software for data acquisition. The average values for each set of duplicate samples or standards were within 15% of the mean. All the analytes in serum samples were determined by comparing the mean of duplicate samples with the standard curve for each assay. 

### 2.4. Alkaline Phosphatase

Alkaline phosphatase (AP) was measured using an Alkaline phosphatase p-Nitrophenylphosphate. Kinetic DGKC (Spinreact, Barcelona, Spain) is used, in which the rate of p-Nitrophenol formation is proportional to the catalytic concentration of alkaline phosphatase present in the sample, and spectrophotometrically measured (Thermo Spectronic, Rochester, NY, USA) at 405 nm wavelength.

### 2.5. Total Antioxidant Capacity (TAC)

The determination of the antioxidant capacity in plasma was determined using ABTS following the method described by Re et al. (1999) [29]. A 7 mM ABTS stock solution (2,2’-Azino-Bis 3-ethylbenzothiazoline6-sulfonic acid diammonium salt) was prepared and left stirring for 19 h, preserving it from light. A 7 mM Trolox solution (6-hydroxy-2,5,7,8 tetramethylchroman-2-carbonate 97%) (5 mL) and PBS 1:10 was also prepared. Plasma samples were diluted 1:10. Then, 1 mL of ABTS was mixed with 43 mL of PBS, and 196 µL of ABTS and 4 µL were added per well. A blank was added in duplicate 4 µL PBS per well. The standard curve was measured in triplicate (Trolox: Ethanol. 0, 125, 250, 500, 750, 1000), and finally, the sample was spectrophotometrically measured (Thermo Spectronic, Rochester, NY, USA) at a 734 nm wavelength.

### 2.6. 8-Hydroxy-2’-deoxyguanosine (8-OHdG)

To measure 8-hydroxy-2′-deoxyguanosine (8-OHdG), an in vitro enzyme-linked immunosorbent assay (ELISA), for the quantitative detection of the oxidative DNA adduct, 8-OHdG was used. To eliminate substances that could interfere with the reaction, the serum was filtered using an ultra-filter (a cut-off molecular weight of 10,000). Results were read at 450 nm on a microplate reader (Bio-tek, Winooski, VT, USA).

### 2.7. Thiobarbituric Acid–Reactive Substances (TBARS) Measurement

Lipid peroxidation was measured on plasma by assessing the concentration of thiobarbituric acid–reactive substances (TBARS). A fraction of plasma (0.5 mL) was mixed with 1 mL of 15% trichloroacetic acid (Sigma-Aldrich, Burlington, MA, USA) and centrifuged at 80× *g* for 10 min. One ml of supernatant was mixed with 1 mL of TBA reagent (0.67%) and the mixture was kept in a boiling water bath for 20 min. The reaction product was extracted and measured by spectrophotometric analysis (Thermo Spectronic, Waltham, MA, USA) at 532 nm. The assay procedure was calibrated using tetraethoxypropanone (Sigma-Aldrich, St. Louis, MO, USA) as a malondialdehyde source.

### 2.8. Statistical Analysis

All variables were checked for normality and homogeneity of variance using the Kolmogorov–Smirnoff and Levene tests, respectively. Categorical variables were compared with the chi-square test. To evaluate the existence of significant differences between groups (control vs. progesterone), a T-Student test was performed for unpaired samples, in the case of samples following normality, and a Mann–Whitney U test was used for samples not following normality. To evaluate the existence of significant differences between the different sampling in each group (T1 vs. T3), a T-Student test was used for paired samples, in case of samples following normality and a Wilcoxon test for samples not following normality. Data are presented as Mean ± Standard Error of Mean. To assess the relationships between the variables of interest (gestational age at delivery, preeclampsia, preterm birth <37 weeks, weight at birth, neonatal therapy, and morbidity) and the biomarkers studied, logistic regression was performed for each parameter, except for gestational age at birth and birth weight, which were analyzed using linear regression. The reported results include odds ratios or estimates, along with their corresponding 95% confidence intervals (CI) and *p*-values. The significance threshold has been adjusted using the FDR method (False Discovery Rate). For all statistical analyses, a value of *p* < 0.05 was considered statistically significant. All statistical analyses were performed using R statistical software version 4.3.0 (R Core Team (2023), https://www.r-project.org/).

## 3. Results

### 3.1. Maternal–Neonatal Characteristics and Clinical Outcomes

Baseline maternal characteristics are shown in Table 1. No statistically significant differences were found between the two study groups in any of the parameters shown. Table 2 shows the fetal and neonatal baseline characteristics of the twins participating in the study, as well as the main clinical outcomes considered. Similarly, no statistically significant differences were identified for any of the parameters assessed.

### 3.2. Biomarkers of Bone Turnover Studied

**Figure 2:**
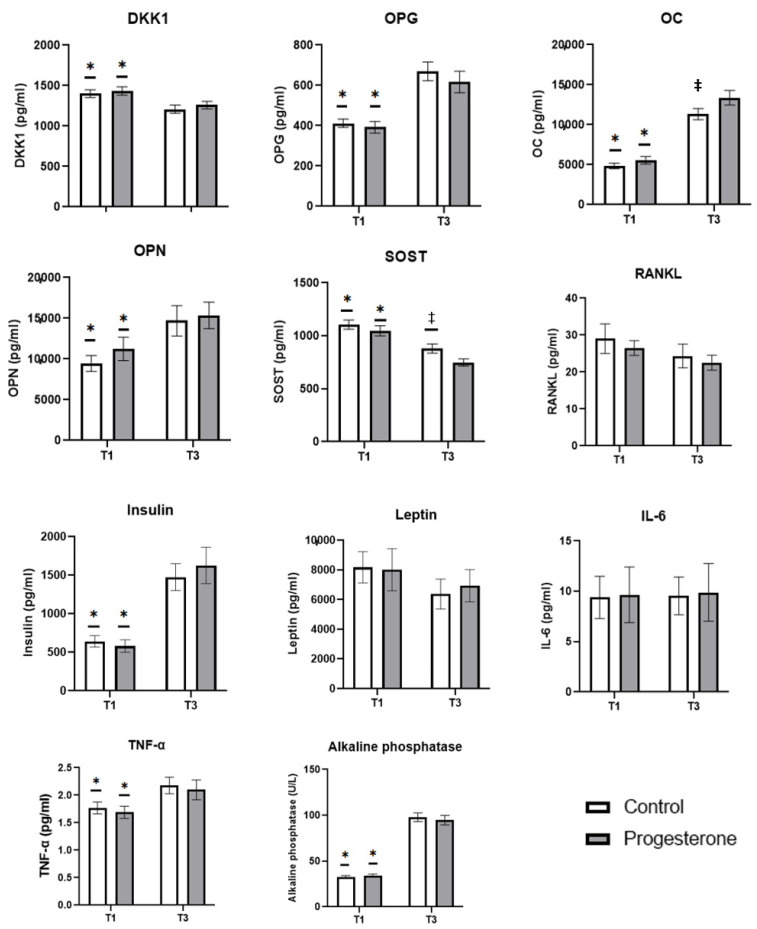
The effect of progesterone administration on maternal serum bone biomarkers values. Data are presented as mean ± standard error of mean. *: indicates the existence of statistically significant differences in each group between the two samples obtained (T1 and T3) (*p* < 0.05); ‡: indicates the existence of statistically significant differences between groups (*p* < 0.05). T1: First trimester; T3: third trimester.

The biomarkers related to maternal bone metabolism studied are shown in Figure 2. Both groups showed a similar evolution in these biomarkers throughout gestation. A gestation-associated decrease in DKK1 and SOST values was observed, with statistically significant differences (*p* < 0.05) between the concentrations found in the first-trimester sampling (T1) and that obtained in the third trimester (T3). In contrast, a gestation-associated increase was observed in the concentrations of OPG, OC, OPN, alkaline phosphatase, insulin, and TNF-α, with statistically significant differences (*p* < 0.05) between T1 and T3. RANKL, leptin, and IL-6, did not show changes throughout gestation in any of the study groups. Results obtained show that the administration of progesterone during the twin gestation process increases maternal serum osteocalcin concentration in the third trimester, with statistically significant differences (*p* < 0.05) between the control group and the progesterone group; on the contrary, this administration decreases SOST concentration in the same trimester, with statistically significant differences (*p* < 0.05), between both groups.

### 3.3. Biomarkers of Oxidative Stress Studied

**Figure 3:**
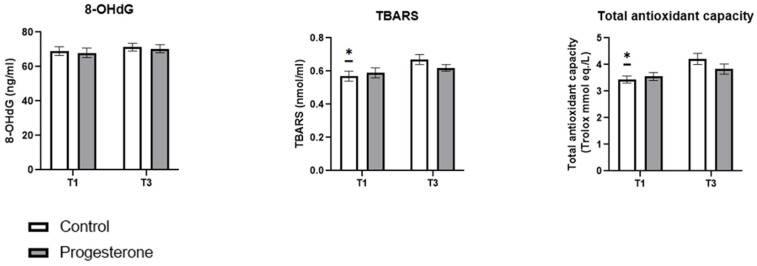
The effect of progesterone administration on maternal oxidative stress. Data are presented as mean ± standard error of mean. *: indicates the existence of statistically significant differences in each group between the two samples obtained (T1 and T3) (*p* < 0.05). T1: First trimester; T3: third trimester.

The oxidative stress markers studied are shown in Figure 3. While no statistically significant differences were observed between the study groups at any specific time point, notable differences were identified in the progression of some biomarkers during pregnancy. In the placebo group, there was a statistically significant increase (*p* < 0.05) in maternal serum concentrations of TBARS and the total maternal plasma antioxidative capacity between T1 and T3. This increase was not observed in the progesterone group. Additionally, no changes in maternal serum 8-OHdG concentrations were detected in either the placebo or the progesterone group throughout the study period.

### 3.4. Regressions Between Maternal and Neonatal Variables of Interest and the Biomarkers Studied

Table S1 (Supplementary Materials) shows all the results obtained in the statistical analysis used to study the relationship between the clinical outcomes of interest studied (gestational age at delivery, preeclampsia, preterm birth <37 weeks, weight at birth, neonatal therapy, and morbidity) and the biomarkers analyzed, adjusted by group.

In relation to gestational age at delivery, a statistically significant negative relationship (Estimate = −0.002, 95% CI = −0.003–−0.000, *p* = 0.015) was observed with maternal serum SOST values in the third trimester and with maternal serum TBARS values in the third trimester (Estimate = −1.946, 95% CI (−3.652–−0.241), *p* = 0.038). A statistically significant negative relationship (Estimate = −0.335, 95% CI (−0.629–−0.042), *p* = 0.038) was also observed between maternal serum SOST values in the third trimester and birth weight, as well as with maternal serum OPN values in the third trimester (Estimate = −0.007, 95% CI (−0.013–−0.001), *p* = 0.047).

In relation to preterm birth before week 37, a negative relationship was observed with the serum values of osteocalcin in the first trimester (Odds ratio = 1.000, 95% CI (1.000–−1.000), *p* = 0.040) and with third-trimester maternal serum sclerontin values (Odds ratio = 1.002, 95% CI (1.000–−1.004), *p* = 0.044).

Finally, a statistically significant negative relationship (Odds ratio = 1.004, 95% CI (1.001–1.006), *p* = 0.008) was observed between SOST T3 and the presence of preeclampsia and a statistically significant positive relationship (Odds ratio = 0.002, 95% CI (0.001–0.004), *p* = 0.020) between the alkaline phosphatase concentration in the third trimester and the neonatal therapy and morbidity variable.

## 4. Discussion

To the best of our knowledge, this is the first study designed to assess how progesterone administration can influence bone turnover and oxidative damage during twin gestation. Twin pregnancy shows a higher incidence of prematurity [6,7]. In an attempt to reduce this incidence, progesterone administration has been used, among other interventions [8], with sufficient evidence highlighting that this hormone is able to decrease the incidence of preterm birth, although mainly in singleton pregnancies [11], showing the results in twin pregnancies more controversy [8,15]. However, progesterone has multiple physiological functions [9] that could influence other processes of importance in maternal, fetal and neonatal health, such as bone turnover or oxidative stress.

Gestation is characterized by high maternal bone turnover in order to ensure adequate mineralization and bone genesis in the fetus, especially during the third trimester [19]. This high turnover could cause maternal bone deterioration, especially when it comes to a twin pregnancy, although there are compensatory mechanisms to avoid this possible maternal damage [18]. Throughout gestation, an increase in intestinal calcium absorption is observed, as well as an increase in the rate of bone resorption and an increase in the rate of bone formation [30,31]. In this sense, the first two trimesters of pregnancy are marked by strong bone resorption and, until the third trimester, an increase in formation biomarkers is not observed [32]. This behaviour is observed in our study; the results show that, in the first trimester, biomarkers related to bone turnover favour the resorption process (higher concentrations of DKK1 and SOST and lower concentrations of OPG, OC, and alkaline phosphatase) over bone formation (lower concentrations of OPN, insulin, and TNF-α), with the opposite being observed in the third trimester, where the bone formation process is more favoured (lower concentrations of DKK1 and SOST and higher concentrations of OPG, OC, and alkaline phosphatase) than that of resorption (higher concentrations of OPN, insulin, and TNF-α).

This increase in bone formation during the third trimester coincides with the trimester of greatest maternal calcium transfer to the fetus [31], which may seem paradoxical. Nevertheless, bone resorption and formation are coupled, so that, after a period of resorption (first and second trimester), an increased period of formation appears, which is delayed in gestation [33,34]. Furthermore, some authors consider that the behaviour of some biomarkers favouring bone formation, such as OPG, could be a compensatory mechanism that could prevent excessive maternal bone resorption and protect the maternal skeleton [35].

As mentioned, along with this effect of progesterone on premature birth, other processes, that have not been studied, may be affected, especially in twin pregnancy [11]. One of these processes could be maternal bone turnover. In spite of some controversy, this steroid hormone shows a protective effect on bone tissue in certain pathologies, favouring bone formation processes [24,25]. In our study, the vaginal administration of 600 mg per day of progesterone, presents a small effect on the maternal bone turnover process since it affects only two of the biomarkers studied, increasing the concentration in the third trimester of osteocalcin and decreasing that of sclerontin, which would indicate an augmentation in bone formation processes in this third trimester.

Osteocalcin is a biomarker of bone formation, whose implications as a hormone linking bone metabolism with energy metabolism are currently noteworthy, featuring a key role as a regulator of energy and glucose homeostasis [36]. There are few examples of data, and these being contradictory, in relation to the effect of progesterone on this biomarker, observing a dose-dependent increase in the expression of osteocalcin mRNA after the administration of progesterone [37] but also the absence of a link between progesterone administration and osteocalcin concentrations [38]. The few existing studies have been carried out in cell cultures or non-pregnant women, so this is the first study showing an effect of progesterone on osteocalcin concentration in twin pregnancies. The second biomarker affected is sclerontin, a potent inhibitor of bone formation by stimulating the resorption process through inhibition of the physiological Wnt signalling pathway and decreasing osteoprogesterin expression [39]. The administration of progesterone affects the concentration of sclerontin in the third trimester, showing a lower concentration than the control group, which would indicate a positive effect on bone formation.

The clinical repercussions of the positive effect of progesterone administration on the bone formation process in the third trimester cannot be exactly known. If the hypothesis of a compensatory mechanism to prevent excessive maternal bone resorption is considered [35], the maternal skeleton would be protected, but at the same time, increased maternal bone formation could lead to a decrease in the resorption and transfer of calcium to the fetus, which, although it would protect the mother, could harm the development of the bones of the fetus. Therefore, further studies are needed to understand the impact of these results on maternal and fetal bone health.

Regarding sclerontin, several studies have shown a relationship between maternal levels of this biomarker in the second trimester, fetal abdominal circumference, and subcutaneous fat deposition, which would contribute to higher birth weight, even though the association has not been observed in other studies [40]. In the present investigation, and as a secondary outcome, the existence of relationships between the biomarkers studied and the main maternal and neonatal clinical outcomes was research, and among them, those observed for sclerontin after adjustment by the group should be highlighted. A negative relationship has been observed between sclerontin levels in the third trimester with birth weight and gestational age and a positive relationship with preterm birth <37 weeks. This association could indicate a mild effect of sclerontin on prematurity. If the effect of progesterone on this bone biomarker is considered, it would be interesting to investigate whether this could be a mechanism of action of progesterone in the prevention of preterm birth.

As previously mentioned, another factor to consider in relation to bone turnover is oxidative stress, which increases during pregnancy and has been shown to negatively affect bone tissue by increasing resorption processes by osteoclasts and inhibiting osteoblast differentiation [41]. Several studies have shown an evolution of oxidative aggression parallel to the gestation evolution, observing a rise in oxidative damage markers and antioxidant defence mechanisms in the third trimester with respect to those observed in the first trimester [23,42]. There are multiple factors that can trigger the increase in oxidative stress observed during pregnancy, including an increase in inflammatory signalling, as observed in our study by the increase in TNF-α levels in the third trimester compared to the first trimester of pregnancy, which are directly related to oxidative stress, tissue destruction, and molecular damage [43]. In our study, in relation to the biomarkers of oxidative stress studied, a similar behaviour has been observed in the results of this study, although only in the control group. The group administered with progesterone did not show such an increase in the lipoperoxidation biomarker, nor in the total antioxidative capacity, which could indicate an antioxidant protective effect of progesterone. This antioxidant effect of progesterone has been observed in other studies [17], although this is the first one to show this result in pregnant women.

As has been commented, to our knowledge, this is the first study to evaluate the effect of vaginal administration of progesterone in twin pregnancies on maternal bone turnover and oxidative damage. However, it is important to indicate that the study showed some methodological limitations that will be considered in subsequent studies: First, although all blood samples were collected under identical conditions, immediately processed in situ and snap frozen, allowing all the samples to have the same background oxidative damage and they are comparable. This process could have been improved by using some preservatives to collect blood samples for oxidative stress detection to avoid possible post hoc effects. Secondly, although the biomarkers used as indicators of oxidative damage in this study are still relevant and validated in the scientific literature, the use of additional, more specific and less generic biomarkers, could have allowed us to obtain a more complete view of oxidative stress suffered by these mothers during twin pregnancy. However, in this study, due to sample availability and economic reasons, because this study is part of a multicentric study, this was not possible. Finally, it is important to highlight that several factors that could be a source of bias in this type of study, such as diet and physical activity, has not been taken into account.

## 5. Conclusions

The present study shows, for the first time, that the vaginal administration of 600 mg per day of progesterone in twin pregnancies influences maternal bone turnover, slightly increasing bone formation in the third trimester of gestation. In addition, it shows, also for the first time, an antioxidant effect of this steroid hormone in this type of pregnancy. Despite these results, more studies are necessary to delve into the maternal, fetal and neonatal clinical benefits or harms of this administration.

## Figures and Tables

**Table 1:** Baseline maternal characteristics.

		Control	Progesterone
Maternal age (years)		34.6 ± 0.7	33.9 ± 0.8
Height (cm)		165.0 ± 1.0	164.0 ± 0.9
Weight (kg)		69.8 ± 2.0	69.4 ± 2.2
BMI (kg/m^2^)		25.5 ± 0.7	25.7 ± 0.7
Conception	In vitro fertilization	16 (31.4%)	13 (28.3%)
	Ovulation drugs	1 (2.0%)	1 (2.2%)
	Natural	34 (66.7%)	32 (69.6%)
Parity	Nulliparous	25 (49.0%)	24 (52.1%)
	Multiparous	26 (51.0%)	23 (47.9%)
Chorion	Dichorionic	42 (82.4%)	40 (87.0%)
	Monochorionic	9 (17.6%)	6 (13.0%)
Cervical Length (mm)		36.7 ± 0.9	36.2 ± 0.8
Preeclampsia	No	46 (90.2%)	42 (91.3%)
	Yes	5 (9.8%)	4 (8.7%)
Preterm Delivery (<37 weeks)	No	30 (58.8%)	25 (54.3%)
	Yes	21 (41.2%)	21 (45.6%)

Values are means ± standard error of mean or n (%) as appropriate.

**Table 2:** Twin baseline characteristics.

		Control		Progesterone	
		Twin 1	Twin 2	Twin 1	Twin 2
Length of largest fetus (week 12)		13.2 ± 0.7		13.1 ± 0.7	
Cranial–caudal length		68.8 ± 1.4	68.8 ± 1.4	67.8 ± 1.5	68.0 ± 1.4
Gestational age (week)		36.9 ± 0.2		36.5 ± 0.3	
Birth weight (g)		2537.6 ± 58.1	2537.6 ± 58.1	2426.9 ± 65.1	2398.4 ± 67.9
Neonatal Therapy	Admission to NICU	3 (6.0%)	2 (4.0%)	4 (8.0%)	3 (6.0%)
	Need for ventilation	5 (10.0%)	4 (8.0%)	5 (10.0%)	5 (10.0%)
Neonatal Morbidity		1 (2.0%)	1 (2.0%)	1 (2.0%)	1 (2.0%)

Values are means ± standard error of mean or n (%) as appropriate. Neonatal morbidity: respiratory distress syndrome, intraventricular hemorrhage, anemia, necrotizing enterocolitis, retinopathy, sepsis.

## Data Availability

The raw data supporting the conclusions of this article will be made available by the authors, without undue reservation.

## Supplementary Materials

The following supporting information can be downloaded at https://www.mdpi.com/article/10.3390/antiox14030324/s1, Table S1: Regression model adjusted by the treatment group that compares the biochemical variables studied with the maternal and neonatal clinical outcomes of interest studied.
