# Supplementary material for: Vaginal Administration of Progesterone in Twin Gestation: Influence on Bone Turnover and Oxidative Stress
Source: Antioxidants (Basel). 2025 Mar 8;14(3):324. doi: 10.3390/antiox14030324 (PMC11939192; doi:10.3390/antiox14030324)
Supplement: Supplementary file 1 [file antioxidants-14-00324-s001.zip › antioxidants-3370245-supplementary.pdf]

Table S1: Regression model adjusted by the treatment group that compares the biochemical variables studied with the maternal and neonatal clinical outcomes of interest studied.

	Gestational age at birth		Preeclampsia		Preterm (week 37th)		Weight at birth		Neonatal therapy and morbidity	
	Estimate (95% CI)	P *Value*	Odds ratio (95% CI)	P *Value*	Odds ratio (95% CI)	P *Value*	Estimate (95% CI)	P *Value*	Odds ratio (95% CI)	P *Value*
DKK1 (T1)	-0.001 (-0.002 – 0.000)	0.279	1.001 (0.999 – 1.003)	0.474	1.000 (0.999 – 1.001)	0.673	-0.153 (-0.381 – 0.076)	0.285	0.000 (-0.000 – 0.000)	0.827
DKK1 (T3)	-0.001 (-0.002 – 0.000)	0.247	1.001 (0.999 – 1.003)	0.334	1.001 (1.000 – 1.002)	0.312	-0.076 (-0.306 – 0.155)	0.520	0.000 (-0.000 – 0.000)	0.993
OPG (T1)	-0.001 (-0.003 – 0.001)	0.223	1.003 (0.999 – 1.006)	0.146	1.001 (0.998 – 1.003)	0.629	-0.347 (-0.798 – 0.103)	0.196	-0.000 (-0.000 – 0.000)	0.689
OPG (T3)	-0.000(-0.001 – 0.000)	0.336	1.001 (1.000 – 1.003)	0.145	1.001 (1.000 – 1.002)	0.377	-0.051 (-0.279 – 0.177)	0.661	0.000 (-0.000 – 0.000)	0.624
OC (T1)	-0.000 (-0.000 – 0.000)	0.452	1.000 (1.000 – 1.000)	0.272	***1.000 (1.000 – 1.000)***	***0.040***	-0.002 (-0.029 – 0.025)	0.889	-0.000 (-0.000 – 0.000)	0.602
OC (T3)	-0.000(-0.000 – 0.000)	0.238	1.000 (1.000 – 1.000)	0.593	1.000 (1.000 – 1.000)	0.206	-0.001 (-0.017 – 0.016)	0.937	0.000 (-0.000 – 0.000)	0.732
OPN (T1)	0.000(-0.000 – 0.000)	0.911	1.000 (1.000 – 1.000)	0.865	1.000 (1.000 – 1.000)	0.906	-0.005 (-0.014 – 0.004)	0.315	0.000 (-0.000 – 0.000)	0.654
OPN (T3)	-0.000(-0.000 – 0.000)	0.856	1.000 (1.000 – 1.000)	0.846	1.000 (1.000 – 1.000)	0.712	***-0.007 (-0.013 – -0.001)***	***0.047***	0.000 (-0.000 – 0.000)	0.619
SOST (T1)	-0.001(-0.002 – 0.000)	0.209	1.002 (0.999 – 1.004)	0.234	1.000 (0.999 – 1.002)	0.620	-0.128 (-0.374 – 0.119)	0.311	-0.000 (-0.000 – 0.000)	0.838
SOST (T3)	***-0.002(-0.003 – -0.000)***	***0.015***	***1.004 (1.001 – 1.006)***	***0.008***	***1.002 (1.000 – 1.004)***	***0.044***	***-0.335 (-0.629 – -0.042)***	***0.038***	0.000 (-0.000 – 0.000)	0.391
RANKL (T1)	0.007 (-0.007 – 0.021)	0.349	0.974 (0.878 – 1.016)	0.733	0.992 (0.966 – 1.011)	0.733	2.139 (-1.321 – 5.599)	0.303	-0.001 (-0.003 – 0.002)	0.679
RANKL (T3)	0.004 (-0.014 – 0.021)	0.687	0.970 (0.854 – 1.019)	0.762	0.992 (0.960 – 1.014)	0.705	0.814 (-3.326 – 4.953)	0.700	-0.000 (-0.003 – 0.003)	0.982
AP (T1)	-0.023 (-0.057 – 0.011)	0.284	0.975 (0.887 – 1.050)	0.849	1.003 (0.959 – 1.048)	0.892	-3.223 (-11.609 – 5.163)	0.451	0.003 (-0.004 – 0.010)	0.948
AP (T3)	-0.007 (-0.017 – 0.002)	0.176	1.018 (1.002 – 1.036)	0.044	1.005 (0.993 – 1.018)	0.580	-2.361 (-4.590 – -0.132)	0.057	***0.002 (0.001 – 0.004)***	***0.020***
Insulin (T1)	-0.000 (-0.001 – 0.000)	0.651	1.001 (1.000 – 1.002)	0.286	1.000 (1.000 – 1.001)	0.629	0.035 (-0.111 – 0.180)	0.639	-0.000 (-0.000 – 0.000)	0.642
Insulin (T3)	-0.000 (-0.000 – 0.000)	0.849	0.999 (0.998 – 1.000)	0.105	1.000 (1.000 – 1.000)	0.909	0.024 (-0.031 – 0.079)	0.399	0.000 (-0.000 – 0.000)	0.652
Leptin (T1)	-0.000 (-0.000 – 0.000)	0.759	1.000 (1.000 – 1.000)	0.139	1.000 (1.000 – 1.000)	0.736	0.005 (-0.004 – 0.014)	0.306	-0.000 (-0.000 – 0.000)	0.748
Leptin (T3)	-0.000 (-0.000 – 0.000)	0.271	1.000 (1.000 – 1.000)	0.843	1.000 (1.000 – 1.000)	0.411	-0.001 (-0.015 – 0.013)	0.865	-0.000 (-0.000 – 0.000)	0.609
TNF-α (T1)	0.064 (-0.365 – 0.493)	0.770	2.146 (0.986 – 4.648)	0.069	1.147 (0.669 – 1.980)	0.637	0.122 (-103.81 – 104.05)	0.998	-0.036 (-0.006 – 0.002)	0.603
TNF-α (T3)	-0.053 (-0.343 – 0.237)	0.722	1.056 (0.530 – 1.818)	0.856	1.010 (0.698 – 1.452)	0.956	-36.803 (-106.66– 33.061)	0.302	0.003 (-0.006 – 0.011)	0.291
Il-6 (T1)	0.007 (-0.014 – 0.027)	0.526	0.991 (0.914 – 1.030)	0.802	0.997 (0.969 – 1.022)	0.787	2.781 (-2.046 – 7.608)	0.350	-0.002 (-0.121 – 0.048)	0.638
IL-6 (T3)	0.007 (-0.013 – 0.027)	0.509	0.991 (0.913 – 1.030)	0.824	0.995 (0.939 – 1.051)	0.981	2.226 (-2.616 – 7.048)	0.369	0.048 (-0.009 – 0.105)	0.693
8-OHdG (T1)	-0.018(-0.035 – -0.000)	0.071	1.033 (0.992 – 1.083)	0.203	1.016 (0.994 – 1.041)	0.242	-4.090 (-8.327 – 0.146)	0.088	0.002 (-0.002 – 0.005)	0.857
8-OHdG (T3)	-0.019 (-0.039 – 0.001)	0.101	1.049 (1.000 – 1.111)	0.106	1.024 (0.997 – 1.052)	0.127	-4.217 (-9.100 – 0.666)	0.136	0.002 (-0.003 – 0.006)	0.907
TBARS (T1)	-0.669 (-2.518 – 1.179)	0.478	10.824(0.319 – 80.74)	0.231	6.210 (0.608 – 76.900)	0.199	-14.345(-462.954 – 34.26)	0.950	-0.014 (-0.381 – 0.353)	0.941
TBARS (T3)	***-1.946 (-3.652 – -0.241)***	***0.038***	0.375 (0.003 – 13.446)	0.801	10.408 (1.091 – 134.965)	0.079	-12.352(-546.414–299.37)	0.567	0.227 (-0.116 – 0.570)	0.585
TAC (T1)	-0.146 (-0.479 – 0.186)	0.388	1.092 (0.544 – 2.376)	0.840	1.108 (0.731 – 1.695)	0.677	-26.092(-106.716 – 54.53)	0.526	0.031 (-0.035 – 0.097)	0.946
TAC (T3)	-0.022 (-0.258 – 0.214)	0.855	0.757 (0.346 – 1.301)	0.624	0.978 (0.719 – 1.312)	0.880	44.470 (-12.066 – 101.006)	0.185	0.001 (-0.046 – 0.048)	0.967

The significance threshold has been adjusted using the FDR method (False Discovery Rate). differences between the value of the biochemical variable in the first and third trimester of pregnancy are shown with the values in bold type ($p<0.05$)
